# An interview with Ondřej Strýček, 2025 Epilepsia Open prize winner for clinical research

**DOI:** 10.1002/epi4.70040

**Published:** 2025-05-19

**Authors:** Merab Kokaia, Piero Perucca

**Affiliations:** ^1^ Faculty of Medicine, Epilepsy Center Lund University Lund Sweden; ^2^ Department of Medicine (Austin Health) The University of Melbourne Melbourne Victoria Australia; ^3^ Bladin‐Berkovic Comprehensive Epilepsy Program Austin Health Melbourne Victoria Australia

**Figure:**
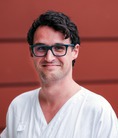
Ondřej Strýček

## 1 TELL US ABOUT YOURSELF

I graduated with an MD from Charles University in Prague and later earned a PhD in neurology from Masaryk University in Brno, where my research focused on functional connectivity in temporal lobe epilepsy. During my PhD studies, I joined the First Department of Neurology at St. Anne's University Hospital in Brno, part of the Brno Epilepsy Center. Currently, one of my main research directions is the application of multimodal approaches in epilepsy, particularly in the identification of epileptogenic lesions and predicting postoperative outcomes. Clinically, I specialize in epileptology and the presurgical evaluation of patients undergoing epilepsy surgery.

## 2 HOW DID YOU BECOME INTERESTED IN CONDUCTING RESEARCH IN THIS FIELD?

My colleague, Martin Kojan, PET imaging expert and a coauthor of this study, introduced me to a new methodology—metabolic connectivity (MC) analysis. I would like to take this opportunity to extend my gratitude to him here, as well as to the other coauthors, for their contributions to this research. MC is a novel technique based on PET that reflects the coordinated functional organization of the brain based on interregional metabolic dependencies. I found the concept of MC interesting, as it does not represent true connectivity in the classical sense but rather reflects correlations in metabolic activity between different brain regions, offering a complementary perspective on brain network organization.

Recognizing its potential to complement conventional structural and electrophysiological modalities, I became particularly interested in its prospective role in presurgical evaluation and optimizing patient selection for epilepsy surgery. Furthermore, I saw the opportunity for PET data, routinely used in clinical practice, to provide additional insights beyond its traditional applications in epilepsy diagnostics.

## 3 PLEASE EXPLAIN THE QUESTION YOUR STUDY ADDRESSED, AND HOW YOU DESIGNED IT

Our study aimed to determine whether MC patterns derived from [18 F]‐fluorodeoxyglucose (FDG)‐PET scans could serve as biomarkers for predicting surgical outcomes in mesial temporal lobe epilepsy (MTLE).

Because the Brno Epilepsy Center has a large cohort of surgically treated epilepsy patients, we aimed to investigate why epilepsy surgery fails in certain cases. We specifically focused on hippocampal sclerosis, a well‐defined form of drug‐resistant epilepsy with uniform pathology and localization, making it an ideal model for our pilot study. Furthermore, we aimed to evaluate the applicability of this method in our patient cohort and assess whether its findings could offer a potential clinical implementation.

First, we compared MC of patients with MTLE to that of healthy controls (HC) to better understand changes associated with epilepsy. We then analyzed preoperative PET scans of MTLE patients who underwent anterior mesial temporal resection (AMTR), comparing MC differences between those who achieved seizure freedom and those who did not.

## 4 WHAT WERE THE RESULTS AND HOW DO YOU INTERPRET YOUR FINDINGS?

Our study demonstrated metabolic network changes in patients with MTLE compared to HC. These changes were not limited to the lesional hippocampus but extended to other brain regions, suggesting that epilepsy should be conceptualized as a condition involving widespread network dysfunction. This finding further supports the characterization of focal epilepsy as a network disease, aligning with evidence from MRI and EEG functional connectivity studies. MC analysis particularly highlighted altered connectivity in the operculo‐insular regions, which are known to be key areas for mesial temporal seizure propagation. Patients with decreased MC in these regions may be more likely to experience less favorable surgical outcomes, likely because standard AMTR does not sufficiently disrupt this network, leading to surgical failure. In contrast, MC changes in other areas, such as the orbitofrontal and lateral temporal cortex, were more commonly associated with better seizure control after surgery. These findings suggest the presence of an alternative preferential seizure propagation pathway that, when disrupted by surgery, results in better surgical outcomes.

Our findings suggest that MC analysis could serve as an additional tool for refining the prediction of surgical outcomes, particularly in cases where conventional imaging fails to fully account for surgical success or failure. More importantly, MC analysis may help identify patients who are at higher risk of not achieving seizure freedom after AMTR and who might benefit from further presurgical evaluation, such as invasive EEG, before undergoing surgery.

## 5 WHAT ARE THE NEXT STEPS THAT YOU PLAN TO TAKE, AND WHAT ARE YOUR CAREER GOALS?

We are now expanding this methodology to analyze MC in all patients with temporal lobe epilepsy, beyond just those with hippocampal sclerosis. Additionally, we are investigating how MC correlates with cognitive outcomes after surgery. Furthermore, we aim to compare the information obtained from the classical PET approach with the additional insights provided by MC analysis to determine how these methods complement each other in understanding epilepsy networks.

More broadly, my goal was to incorporate network‐based biomarkers into routine epilepsy care, fostering a more personalized and data‐driven approach to epilepsy treatment. By integrating neuroimaging and electrophysiological data, I aim to enhance preoperative risk assessment and surgical decision‐making, particularly for nonlesional epilepsies, where conventional diagnostic methods may be insufficient.

## 6 WHAT DOES THE EPILEPSIA OPEN PRIZE MEAN FOR YOU, YOUR LABORATORY, RESEARCH INSTITUTE, AND YOUR FUTURE?

This award is a significant recognition for our entire team at the Brno Epilepsy Center, affirming that our research is moving in a meaningful direction. It motivates us to continue exploring new multimodal approaches that will not only improve the identification of epileptogenic lesions but also support the development of more effective and individualized treatment strategies for patients with drug‐resistant epilepsy. On a personal level, this recognition inspires me to further advance epilepsy research, offering opportunities to share our findings with the international community and find new collaborations.

We are grateful to *Epilepsia Open* and the ILAE for this recognition, and we look forward to contributing further to epilepsy research.

Read the winning article: Metabolic connectivity as a predictor of surgical outcome in mesial temporal lobe epilepsy

## CONFLICT OF INTEREST STATEMENT

The authors report no competing or financial interests.

## Data Availability

Data sharing is not applicable to this article as no new data were created or analyzed in this study.

